# Neonatal Mass Urine Screening Approach for Early Detection of Mucopolysaccharidoses by UPLC-MS/MS

**DOI:** 10.3390/diagnostics9040195

**Published:** 2019-11-18

**Authors:** Iskren Menkovic, Anne-Sophie Marchand, Michel Boutin, Christiane Auray-Blais

**Affiliations:** Division of Medical Genetics, Department of Pediatrics, Faculty of Medicine & Health Sciences, Université de Sherbrooke, CIUSSS de l’Estrie-CHUS, 3001, 12th Avenue North, Sherbrooke, QC J1H 5N4, Canada; Iskren.Menkovic@USherbrooke.ca (I.M.); Anne-Sophie.Marchand@USherbrooke.ca (A.-S.M.); Michel.Boutin2@USherbrooke.ca (M.B.)

**Keywords:** mucopolysaccharidosis (MPS), newborn screening, heparan sulfate, dermatan sulfate, UPLC-MS/MS, dried urine spots (DUS)

## Abstract

Mucopolysaccharidoses (MPSs) are lysosomal storage disorders caused by deficiencies of enzymes involved in the catabolism of glycosaminoglycans (GAGs). Various treatments such as enzyme replacement therapy and/or hematopoietic stem cell transplant are available for MPSs. Early initiation of treatment improves the outcome and delays the onset of symptoms, highlighting the need for newborn screening for MPSs. The main objective of this project was to devise and validate a multiplex urine filter paper method for GAG analysis using a tandem mass spectrometry (MS/MS) approach to screen newborns for MPSs. Eluted urine samples from 21-day-old newborns were evaporated and a methanolysis reaction was performed. Samples were resuspended and analyzed using a UPLC-MS/MS system. A one-minute chromatographic method allowed the absolute quantification of heparan sulfate (HS), dermatan sulfate (DS), and creatinine. Method validation revealed high precision (< 9% relative standard deviation) and accuracy (< 7% bias) for all analytes. The reference values normalized to creatinine obtained by the analysis of five hundred 21-day-old newborn urine samples were 34.6 +/-6.2 mg/mmol of creatinine and 17.3 +/-3.9 mg/mmol of creatinine for HS and DS, respectively. We present a rapid and efficient method for populational newborn urine screening using MS/MS, which could also be applied to high-risk screening.

## 1. Introduction

Mucopolysaccharidoses (MPSs) are lysosomal storage disorders (LSDs) caused by a deficit in the glycosaminoglycan (GAG) catabolism [1]. More specifically, MPS I (Hurler syndrome OMIM 607014, Hurler/Scheie syndrome OMIM 607015 and Scheie syndrome OMIM 607016), MPS II (Hunter syndrome OMIM 309900), MPS III (Sanfilippo A–D syndromes, OMIM 252900, 252920, 252930, 252940), MPS VI (Maroteaux-Lamy syndrome, OMIM 253200), and MPS VII (Sly syndrome, OMIM 253220) are characterized by defects in the degradation of heparan sulfate (HS) and/or dermatan sulfate (DS) leading to the accumulation of these biomarkers in several tissues and biological fluids. GAG storage results in progressive cellular damage [2,3]. The MPSs are characterized by a broad clinical spectrum, ranging from severe neonatal forms to attenuated forms diagnosed later on in adults [4]. Symptoms generally involve organomegaly, umbilical and inguinal hernias, hearing loss, macroglossia, coarse facial features as well as cardiac, respiratory, skeletal and cognitive impairments [5,6]. Enzyme replacement therapy (ERT) and/or hematopoietic stem cell transplant (HSCT) are available for patients with MPS I, II, VI, and VII [6,7,8].

Previous studies revealed that although affected newborns usually do not clearly exhibit signs of the disease, an elevation of GAGs can be measured in human fetuses and placentas [9], suggesting that the disease can be detected before the first clinical manifestations occur. Considering the irreversible nature of organ damages in MPS patients, experts have agreed that an early initiation of treatment may lead to significant delay and/or prevent the onset of clinical signs and improved outcomes [6,10,11,12,13,14,15]. Therefore, it has been recommended that newborns be screened for MPSs in the hopes of identifying them early. Moreover, MPS I was added in 2015 to the Recommended Uniform Screening Panel for the United States [10,11,12,13,14,15].

Newborn screening (NBS) methods for LSDs usually involve dried blood spots (DBS) in which the enzyme activity is measured [16,17]. While DBSs can be a reliable matrix for NBS, urine samples offer major advantages, primarily non-invasive specimen collection, particularly in children, which can be performed at home by parents and has easy shipping of samples by regular mail in an envelope. The Provincial Neonatal Urine Screening Program in Sherbrooke, Quebec, Canada is a unique program (voluntary compliance of parents averages nearly 90%) where 21-day-old babies are screened for inherited metabolic disorders using urine samples collected on filter paper (dried urine spots, DUS) and analyzed by thin-layer chromatography. The following disorders are targeted: urea cycle disorders, and organic acidurias [18,19]. More recently, a method allowing the absolute quantification of HS and DS in DUS was developed by our group for high-risk screening using a high-performance liquid chromatography tandem mass spectrometry technology (HPLC-MS/MS) [20]. This analytical method is useful for early detection, as well as monitoring and follow-up of treated patients affected with MPS I, MPS II, MPS VI, and MPS VII. Unfortunately, it was not devised for a large-scale newborn urine screening program such as the one in Quebec (~70,000 newborns/year). However, the method as published, did not include creatinine analysis as part of a multiplex analysis to normalize the GAG levels. The importance of creatinine normalization has been shown to improve urine biomarker evaluation [21]. 

To our knowledge, there is no rapid and non-invasive multiplex method allowing the absolute quantification of GAGs and creatinine using urine samples collected on filter paper, which can be used in a population-based newborn screening setting. The project presented herein had two main objectives: 1) to develop and validate a rapid high-throughput UPLC-MS/MS method suitable for NBS allowing the absolute quantification of HS, DS and creatinine in DUS; and 2) to establish normal reference values using five hundred DUS.

## 2. Materials and Methods

### 2.1. Ethics Approval

Following the validation of the method, anonymized DUS were used to establish reference values before being discarded. After the discussion with the Institutional Review Board from our Institution, it was agreed that this development/validation activity was not considered as “research” according to the article 2.5 of the Canadian TCPS2 (Tri-Council Policy Statement 2), no IRB approval was required.

### 2.2. Urine Specimen Collection on Filter Paper

Five hundred random and anonymized urine samples collected on filter paper (Whatman-GE 903) from 21-day-old newborns with normal amino acid and organic acid profiles were selected as healthy controls and were used to establish normal reference values. All samples were collected and sent by parents by regular postal service. Upon reception, urine filter papers were stored at room temperature until analysis. Similarly, DUS from MPS patients with ages ranging from 1.7 to 10 years old were collected and analyzed for comparison purposes.

### 2.3. Reagents

DS, chondroitin sulfate A (CS), HS, and creatinine standards, as well as methanol-D_4_ (99.8 atom % D) and ammonium acetate (≥ 98% purity) were purchased from Sigma Aldrich (Saint-Louis, MO, USA). Creatinine-D_3_ (methyl D_3_, 99 atom % D) was obtained from CDN Isotopes Inc. (Pointe-Claire, QC, CAN). Optima^®^ LC-MS grade water (H_2_O) was obtained from Fisher Scientific (Fair Lawn, NJ, USA). HPLC grade methanol (MeOH) and LC-MS grade acetonitrile (ACN) were purchased from EMD Chemicals Inc. (Darmstadt, Germany). Methanolic hydrochloric acid (HCl) 3 N was purchased from Oakwood Chemicals (Estill, SC, USA). Acetyl chloride was purchased from Fluka (Milwaukee, WI, USA), ammonium hydroxide (NH_4_OH) was obtained from Fisher Chemicals (Hampton, NH, USA), and formic acid (FA) was from Acros Organics (Thermo Fisher Scientific, Waltham, MA, USA).

#### 2.3.1. Analyte Stock Solutions

DS and HS standards were diluted in H_2_O to make individual stock solutions at 10.0 mg/mL. The deuterated creatinine standard was also diluted in H_2_O to obtain an individual stock solution at 56.6 mg/mL. All standards were kept at 4 °C and were stable for at least one year.

#### 2.3.2. Internal Standard Stock Solutions

Deuterated internal standards (IS) for HS and DS were prepared in-house by deuteriomethanolysis of HS and DS commercial standards, as reported in previous publications [5,20,22]. In summary, a 2.0 M MeOH-D_4_-DCl solution was prepared by adding 80 µL of acetyl chloride dropwise on ice with continuous stirring to 500 μL of MeOH-D_4_. A volume of 580 μL of this 2.0 M MeOH-D_4_-DCl solution was then added to 600 μg of HS or DS. The samples were then vortexed for 30 seconds and incubated for 75 min at 65 °C. Following the incubation, the samples were dried under a stream of nitrogen and resuspended in 1 mL of H_2_O. These stock solutions at a concentration of 600 µg/mL were kept at 4 °C and showed no signs of degradation after 6 months. A stock solution of 20.0 mM of creatinine-D_3_ was prepared for deuterated creatinine. This stock solution was diluted 1:10 in water to obtain a 2.0 mM creatinine-D_3_ solution, which was used for the preparation of the resuspension solution.

#### 2.3.3. Standard Curves and Quality Control Working Solutions

Eight standard solutions with concentrations of 0, 16, 50, 100, 250, 600, 1500, and 2000 µg/mL for HS and DS, and 0, 2.7, 5.4, 6.8, 9.0, 13.6 and 18.1 mg/mL for creatinine were prepared. Also, low, medium and high-quality control (QC) solutions at 75, 200, and 1000 µg/mL of HS and DS with 2.8, 5.7 and 11.3 mg/mL of creatinine, respectively, were prepared from stock solutions by dilution with H_2_O. Both QC and standard solutions were kept at 4 °C and found to be stable for at least 6 months.

#### 2.3.4. Resuspension Solution

The resuspension solution was prepared by mixing 9 mL of ACN, 700 μL of H_2_O, 150 μL of deuterated DS IS stock solution, 50 μL of deuterated HS IS stock solution, and 100 μL of the 2.0 mM creatinine-D_3_ stock solution.

### 2.4. Sample Processing

Filter papers were examined under ultraviolet light to confirm that the amount of urine required for the analysis was sufficient and a 5-cm disk was punched out. For calibration points, 50 μL of each calibration point was added and left to dry at room temperature. The 5-cm disk was then folded in half and deposited in glass bottles. Extraction of the urine sample was done by adding 3.0 mL of 0.01 M NH_4_OH followed by rotary shaking for 10 min (New Brunswick Scientific Shaker, Edison, NJ, USA). One hundred microliters of the extracted urine were transferred into a screw-topped glass tube and evaporated to dryness under a nitrogen stream, followed by a methanolysis reaction as previously described [20]. Briefly, 500 µL of MeOH/HCl 3 N was added to each tube. Samples were vortexed and incubated at 65 °C for 55 min. Following the incubation, samples were once again evaporated to dryness under a nitrogen stream and 200 µL of the resuspension solution was added.

### 2.5. Instrumentation and Parameters

#### 2.5.1. UPLC Parameters

The chromatographic separation of HS, DS, creatinine and their respective deuterated internal standards was performed using a BEH Amide Ultra Performance Liquid Chromatography (UPLC) column (2.1 × 50 mm, 1.7 μm particle size) (Waters Corp. Milford, MA, USA) on an Acquity H-Class UPLC system (Waters Corp., Milford, MA, USA). The method runtime was 1 min with a gradient ranging between 10% and 60% of mobile phase B. Chromatographic parameters are shown in Table 1.

#### 2.5.2. MS/MS Parameters

The quantification of HS and DS-derived disaccharides from the methanolysis reaction, as well as creatinine and their related internal standards, was performed as a multiplex method using a Xevo TQ-S Micro tandem mass spectrometer (Waters). Ionization was performed in positive electrospray ionization (ESI^+^) and the mass spectrometer was operated in multiple reaction monitoring (MRM) mode to ensure that a maximum specificity was achieved. General MS/MS parameters, as well as the transitions used for each of the analytes and their respective deuterated internal standards are shown in Table 2.

#### 2.5.3. Quantification Parameters

The quantification of HS, DS and creatinine was performed using an 8-point calibration curve with the following concentrations: 0, 0.8, 2.5, 5.0, 12.5, 30.0, 75.0 and 100.0 μg/mL for HS and DS and 0, 0.4, 0.8, 2.0, 3.0, 4.0, 6.0, 8.0 mM for the creatinine. Calibration curves were prepared in H_2_O and the resulting linear curves were forced to the origin for all analytes. Data were acquired over a 1-min timeframe. Quantification was done using the response factor and data processing was achieved using TargetLynx® Application Manager, an option with MassLynx™ (version 4.1 SCN810) Software (Waters).

### 2.6. Method Validation

The linearity of the 8-point calibration curve for HS, DS and creatinine was evaluated. Intraday (5 replicates in a day) and interday (5 different days) precision and accuracy assays were evaluated using different concentrations of QC solutions: low (3.8 µg/mL for HS and DS, and 1.3 mM for creatinine), medium (10.0 µg/mL for HS and DS, and 2.5 mM for creatinine), and high (50.0 µg/mL for HS and DS, and 5.0 mM for creatinine). The method accuracy was measured by spiking DUS with 20.0 µL of the previously mentioned QC concentrations. Experimental concentrations were then compared with theoretical values calculated by subtracting endogenous levels of analytes previously measured to the concentration of the spiked QC in order to assess the % bias. All interday analyses for HS, DS and creatinine included an 8-point calibration curve, low, medium and high concentrations (QCs), as well as a control urine sample. Both QC samples and control urine samples were prepared in triplicate for each interday. Adhesion to plastic and glassware was evaluated using QC samples which were transferred 3 times in a glass or plastic insert after the resuspension step. Control samples were used to establish the limit of quantification (LOQ) and the limit of detection (LOD) for all 3 analytes (corresponding to 10× and 3× the standard deviation of a control sample injected 10 times, for LOQ and LOD, respectively). The recovery following the DUS extraction was evaluated by comparing the area under the curve when a 1 mL urine sample was added to a filter paper and then extracted using the elution process described previously versus when a 1.0 mL sample was simply diluted in 3.0 mL of the extraction solution. The freeze/thaw stability of HS, DS and creatinine following the elution step was evaluated by comparing the measured concentration in QC samples before and after 3 freeze/thaw cycles. Finally, the stability of HS, DS, and creatinine in the elution solution was studied for 48 h, one week and one month at temperatures ranging from –30 to 25 °C.

## 3. Results and Discussion

### 3.1. Method Development and Validation

**Figure 1:**
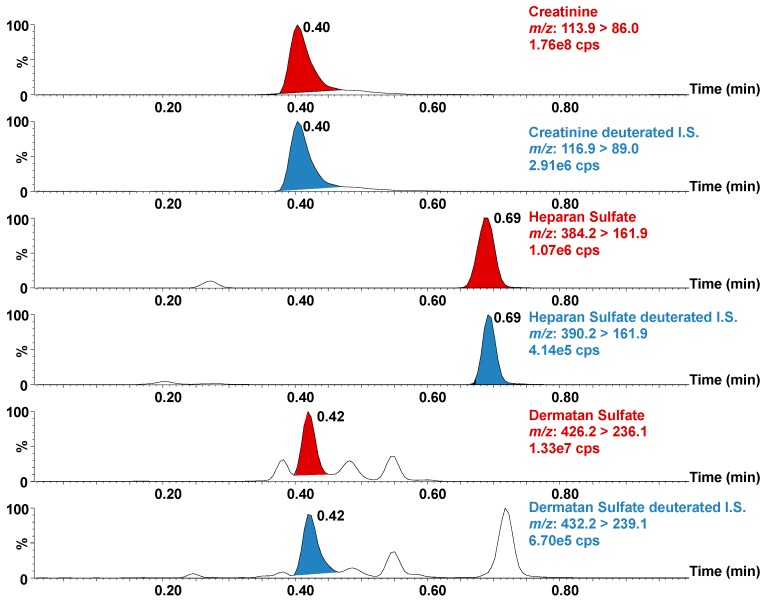
Ion chromatograms of heparan sulfate (HS), dermatan sulfate (DS), and creatinine standards (HS and DS at 34 μg/mL and creatinine at 301 μg/mL) and their deuterated counterparts (deuterated HS at 3.0 μg/mL; deuterated DS at 9.0 μg/mL and deuterated creatinine at 2.3 μg/mL). I.S.: Internal standard; Cps: Counts per second.

The BEH Amide UPLC column allowed a good separation of all analytes based on hydrophilic interactions in a 1-min chromatography run (Figure 1). The peak at 0.56 min was attributed to CS after confirmation with the corresponding standard (60 µg/mL).

Intraday (*n* = 5) and interday (*n* = 5) assays revealed that the biases (%) were less than 7% and the relative standard deviations (RSD %) were less than 9% for HS, DS, and creatinine, respectively. An average coefficient of determination (r^2^) of 0.999 (*n* = 5) was achieved for the calibration curves of all the 3 analytes. There was no evidence of plastic or glassware adsorption (bias < 3%). LOQs were 13.3 µg/mL, 8.2 µg/mL and 0.1 mM for HS, DS, and creatinine, respectively, while LODs were 4.0 µg/mL, 2.5 μg/mL and 0.03 mM for HS, DS, and creatinine, respectively.

Previous stability experiments performed by our group revealed that DS and HS were stable on DUS for 6 weeks at temperatures ranging from 25 to −80 °C [20]. Our results revealed that the creatinine is stable at temperatures ranging from 25 to −30 °C for a 4-week period. Results showed good recoveries at 92% 89% and 94% for HS, DS and creatinine, respectively. Three freeze/thaw cycles had no impact on HS, DS and creatinine levels. Previous results obtained by our group showed that processed samples were stable for at least 48 h at −20 °C, 4 °C and at room temperature (22 °C) [20]. 

### 3.2. Reference Values

**Figure 2:**
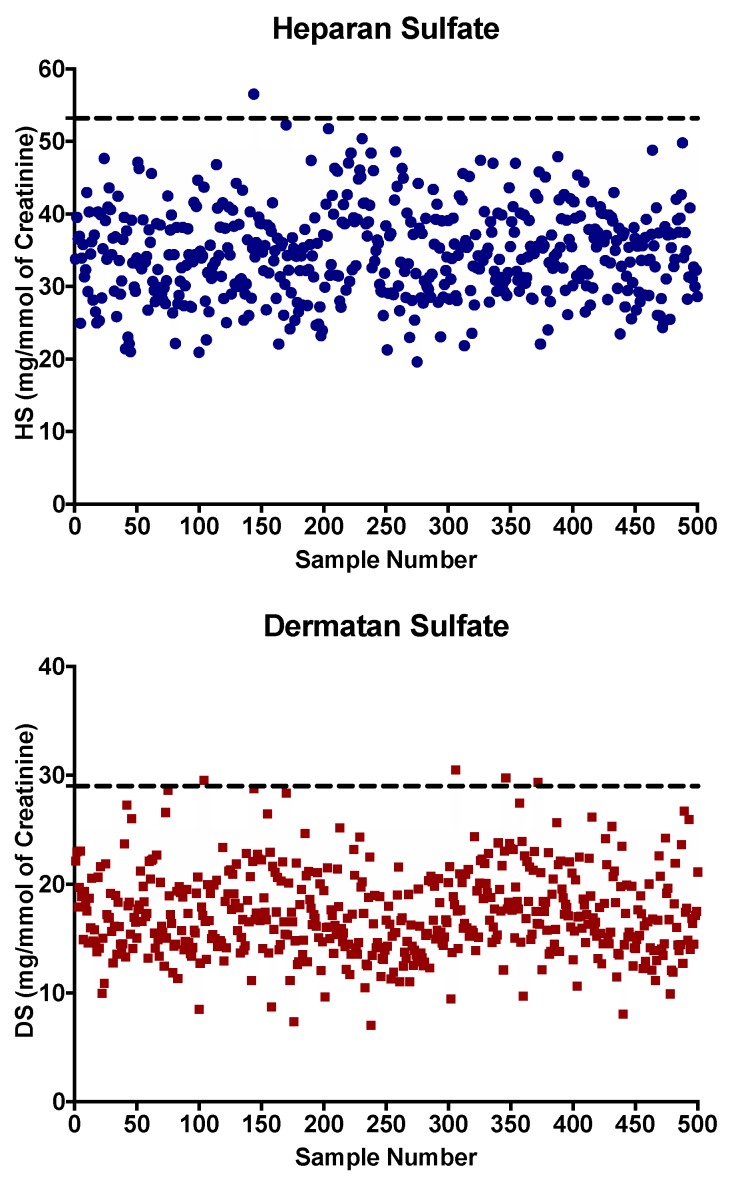
HS and DS concentrations expressed in mg/mmol of creatinine measured in five hundred dried urine spots (DUS) from 21-day-old control newborns. Cutoffs values represented by the dotted line correspond to the average analyte concentration + 3 standard deviations.

Following the method validation, five hundred DUS from 21-day-old control newborns were analyzed to establish reference values. Concentrations measured in DUS varied from 17.9 to 56.5 mg/mmol of creatinine, and 7.3 to 30.5 mg/mmol of creatinine for HS and DS, respectively (Figure 2). We calculated the average + 1 standard deviation (SD) for HS and DS concentrations in 21-day-old control newborns as 34.6 +/− 6.2 mg/mmol of creatinine and 17.3 +/− 3.9 mg/mmol of creatinine, respectively. The limit for the reference value was thus set as the mean + 3 SD with values for HS at 53.2 mg/mmol of creatinine and for DS at 29.0 mg/mmol of creatinine (Figure 2). A larger number of samples will eventually be required to establish reference values in order to reduce the false-positive rate. 

### 3.3. Urine Samples from Confirmed MPS Patients

Urine samples from confirmed MPS patients (Age range: 20 months to 10 years old) were analyzed to evaluate the effectiveness of the proposed multiplex method. Results are shown in Table 3. Unfortunately, DUS from 21-day-old MPS patients were not available. The values measured in 21-day-old control newborns cannot be used to establish reference values for the older MPS patients evaluated because HS and DS urinary levels are known to decrease with age [5]. Reference values for HS and DS in children aged 4–9 years old have been shown to be more than 3 times lower than the 12-month age group [5]. However, DUS from controls with matching age and gender were analyzed to assess the efficiency of the proposed method. As expected, all untreated MPS patients had increased levels of urinary HS and/or DS when compared with matching controls (Table 3); the 3 treated patients (patients 7 to 9) showed slightly increased levels of HS and DS. Unfortunately, based information on the time of initiation of treatment is not available.

## 4. Conclusions

The early identification of MPS affected newborns remains challenging since most of them do not exhibit any signs of the disease early in life [23]. However, considering that several studies demonstrated that GAGs are elevated as early as 21 weeks of gestation [9,24], we strongly believe that newborn screening will be a useful tool to identify patients before the onset of irreversible signs and symptoms associated with the disease. We described herein, the development and validation of a UPLC-MS/MS multiplex method, which allows an absolute quantification of HS, DS and creatinine, and is suitable for newborn screening of MPS I, II, III, VI, and VII using urine DUS from 21-day-old newborns. The proposed method is rapid, efficient, and reliable which allows the analysis of 500 samples per day per UPLC-MS/MS instrument. LC-MS/MS methods for urine GAG quantification have been previously published, but none allows the simultaneous quantification of HS, DS and creatinine by a 1-min method. Unlike dried blood spots, DUS collection offers major advantages since it is non-invasive and easy to collect by parents. It also favors storage and shipping of samples to biochemical genetics and metabolic laboratories at low cost by regular mail. Also, previous studies have shown that GAG concentrations are more elevated in urine specimens than other biological fluids such as blood and plasma, thus favoring urine as a better matrix to quantify GAGs for early detection of MPSs [25,26]. A limitation of this study is the unavailability of samples from 21-day-old infants affected with various MPSs. A future perspective involves the comparison of DUS and DBS glycosaminoglycan methods for MPS screening on the same newborn cohort. While this method is intended to screen newborns, it might also be suitable for high-risk screening [27] and monitoring treated MPS patients, as long as appropriate reference age-related values are established.

## Figures and Tables

**Table 1:** Ultra Performance Liquid Chromatography (UPLC) parameters for the analysis of heparan sulfate (HS), dermatan sulfate (DS), and creatinine.

UPLC Parameters	
Column	BEH Amide
ID × Length	2.1 × 50 mm
Particle size	1.7 μm
Column temperature	40 °C
Weak Wash solvent	95:5 ACN:H_2_O + 0.2% FA
Strong Wash solvent	50:50 MeOH:H_2_O
Injection volume	4.0 μL
Injector	Flow-through needle
Autosampler temperature	10 °C
Mobile phase A	95:5 ACN:H_2_O + 0.2% FA + 10 mM CH_3_COONH_4_
Mobile phase B	10:90 ACN:H_2_O + 0.2% FA + 10 mM CH_3_COONH_4_
Flow rate	0.8 mL/min
Gradient (% mobile phase B)	0.0 → 0.1 min: 10% 0.1 → 0.5 min: 10 →60% (Linear gradient) 0.5 → 1.0 min: 10%

**Table 2:** Tandem mass spectrometry (MS/MS) parameters and transitions used for the quantification of DS, HS, creatinine and their respective deuterated internal standards.

**MS/MS Parameters**			
Ionization mode		ESI	
Polarity		Positive	
Acquisition mode		MRM	
Capillary voltage		3.44 kV	
Desolvation temperature		500 °C	
Desolvation gas flow		700 L/h	
Cone gas flow		150 L/h	
Source temperature Dwell time		120 °C 0.05 s	
Span		0 Da	
**Transitions**			
**Compound**	**Transitions (*m/z*)**	**Cone Voltage (V)**	**Collision Energy (V)**
DS	426.2 > 236.1	36	12
DS-D_6_ IS	432.2 > 239.1	36	12
HS	384.2 > 161.9	18	18
HS-D_6_ IS	390.2 > 161.9	18	18
Creatinine	113.9 > 86.0	34	10
Creatinine-D_3_	116.9 > 89.0	34	10

**Table 3:** Analysis of dermatan sulfate (DS) and heparan sulfate (HS) in urine samples collected on filter papers from Mucopolysaccharidose (MPS) patients.

Patient	MPS Type	Gender	Age (Years)	ERT	HS *	DS *	Control HS *	Control DS *
1	I (H)	M	1.7	U	86.4	42.6	8.4	1.7
2	I (H/S)	M	8.0	U	68.7	36.4	9.7	1.4
3	I (H/S)	F	10	U	48.1	24.2	4.0	1.4
4	II	M	3.0	U	150.3	72.6	10.6	1.3
5	II	M	5.0	U	45.4	15.1	9.6	1.7
6	II	M	5.0	U	47.5	14.9	9.4	0.8
7	II	M	5.0	T	13.4	3.8	7.3	2.3
8	II	M	6.0	T	16.3	4.9	5.8	0.8
9	II	M	7.0	T	12.9	5.2	4.8	1.2
10	IIIA	M	3.0	U	142.1	4.0	8.4	1.8
11	IIIB	F	1.8	U	56.5	1.6	10.2	1.6
12	VI	F	10	U	65.4	46.4	7.7	2.9

Gender: Male (M) or Female (F), Enzyme Replacement Therapy (ERT): Untreated patient (U) or treated patient (T). *Expressed in mg/mmol of creatinine.

## References

[1] Palmieri C., Giger U., Wang P., Pizarro M., Shivaprasad H.L. (2015). Pathological and Biochemical Studies of Mucopolysaccharidosis Type IIIB (Sanfilippo Syndrome Type B) in Juvenile Emus (Dromaius novaehollandiae). Vet. Pathol..

[2] Muenzer J. (2011). Overview of the mucopolysaccharidoses. Rheumatology.

[3] Lawrence R., Brown J.R., Lorey F., Dickson P.I., Crawford B.E., Esko J.D. (2014). Glycan-based biomarkers for mucopolysaccharidoses. Mol. Genet. Metab..

[4] Khan S.A., Peracha H., Ballhausen D., Wiesbauer A., Rohrbach M., Gautschi M., Mason R.W., Giugliani R., Suzuki Y., Orii K.E. (2017). Epidemiology of mucopolysaccharidoses. Mol. Genet. Metab..

[5] Auray-Blais C., Lavoie P., Tomatsu S., Valayannopoulos V., Mitchell J.J., Raiman J., Beaudoin M., Maranda B., Clarke J.T. (2016). UPLC-MS/MS detection of disaccharides derived from glycosaminoglycans as biomarkers of mucopolysaccharidoses. Anal. Chim. Acta.

[6] Mitchell J., Berger K.I., Borgo A., Braunlin E.A., Burton B.K., Ghotme K.A.G., Kircher S.G., Molter D., Orchard P.J., Palmer J. (2016). Unique medical issues in adult patients with mucopolysaccharidoses. Eur. J. Intern. Med..

[7] Gaffke L., Pierzynowska K., Piotrowska E., Węgrzyn G. (2017). How close are we to therapies for Sanfilippo disease?. Metab. Brain Dis..

[8] Giugliani R., Federhen A., Rojas M.V., Vieira T., Artigalás O., Pinto L.L., Azevedo A.C., Acosta A., Bonfim C., Lourenço C.M. (2010). Mucopolysaccharidosis I, II, and VI: Brief review and guidelines for treatment. Genet. Mol. Biol..

[9] Kubaski F., Mason R.W., Nakatomi A., Shintaku H., Xie L., Van Vlies N.N., Church H., Giugliani R., Kobayashi H., Yamaguchi S. (2016). Newborn screening for mucopolysaccharidoses: a pilot study of measurement of glycosaminoglycans by tandem mass spectrometry. J. Inherit. Metab. Dis..

[10] Muenzer J. (2014). Early initiation of enzyme replacement therapy for the mucopolysaccharidoses. Mol. Genet. Metab..

[11] Gabrielli O., Clarke L.A., Bruni S., Coppa G.V. (2010). Enzyme-Replacement Therapy in a 5-Month-Old Boy With Attenuated Presymptomatic MPS I: 5-Year Follow-up. Pediatrics.

[12] Clarke L.A., Atherton A.M., Burton B.K., Day-Salvatore D.L., Kaplan P., Leslie N.D., Scott C.R., Stockton D.W., Thomas J.A., Muenzer J. (2017). Mucopolysaccharidosis Type I Newborn Screening: Best Practices for Diagnosis and Management. J. Pediatr..

[13] Grosse S.D., Lam W.K., Wiggins L.D., Kemper A.R. (2017). Cognitive outcomes and age of detection of severe mucopolysaccharidosis type 1. Genet. Med..

[14] Shone S.M. (2019). Newborn Screening Policy Decisions. North Carol. Med. J..

[15] Laraway S., Breen C., Mercer J., Jones S., Wraith J.E. (2013). Does early use of enzyme replacement therapy alter the natural history of mucopolysaccharidosis I? Experience in three siblings. Mol. Genet. Metab..

[16] Chan M.-J., Liao H.-C., Gelb M.H., Chuang C.-K., Liu M.-Y., Chen H.-J., Kao S.-M., Lin H.-Y., Huang Y.-H., Kumar A.B. (2019). Taiwan National Newborn Screening Program by Tandem Mass Spectrometry for Mucopolysaccharidoses Types I, II, and VI. J. Pediatr..

[17] Hopkins P.V., Campbell C., Klug T., Rogers S., Raburn-Miller J., Kiesling J. (2015). Lysosomal Storage Disorder Screening Implementation: Findings from the First Six Months of Full Population Pilot Testing in Missouri. J. Pediatr..

[18] Auray-Blais C., Cyr D., Drouin R. (2007). Quebec neonatal mass urinary screening programme: From micromolecules to macromolecules. J. Inherit. Metab. Dis..

[19] Auray-Blais C., Giguère R., Lemieux B. (2003). Newborn urine screening programme in the province of Quebec: an update of 30 years’ experience. J. Inherit. Metab. Dis..

[20] Auray-Blais C., Lavoie P., Zhang H., Gagnon R., Clarke J.T., Maranda B., Young S.P., An Y., Millington D.S. (2012). An improved method for glycosaminoglycan analysis by LC–MS/MS of urine samples collected on filter paper. Clin. Chim. Acta.

[21] Chen Y., Shen G., Zhang R., He J., Zhang Y., Xu J., Yang W., Chen X., Song Y., Abliz Z. (2013). Combination of Injection Volume Calibration by Creatinine and MS Signals’ Normalization to Overcome Urine Variability in LC-MS-Based Metabolomics Studies. Anal. Chem..

[22] Zhang H., Young S.P., Millington D.S. (2013). Quantification of Glycosaminoglycans in Urine by Isotope-Dilution Liquid Chromatography-Electrospray Ionization Tandem Mass Spectrometry. Curr. Protoc. Hum. Genet..

[23] Clarke L., Ellaway C., Foster H.E., Giugliani R., Goizet C., Goring S., Hawley S., Jurecki E., Khan Z., Lampe C. (2018). Understanding the Early Presentation of Mucopolysaccharidoses Disorders. J. Inborn. Errors Metab. Screen.

[24] Kubaski F., Brusius-Facchin A.C., Mason R.W., Patel P., Burin M.G., Michelin-Tirelli K., Kessler R.G., Bender F., Leistner-Segal S., Moreno C.A. (2017). Elevation of glycosaminoglycans in the amniotic fluid of a fetus with mucopolysaccharidosis VII. Prenat. Diagn..

[25] Khan S.A., Mason R.W., Giugliani R., Orii K., Fukao T., Suzuki Y., Yamaguchi S., Kobayashi H., Orii T., Tomatsu S. (2018). Glycosaminoglycans analysis in blood and urine of patients with mucopolysaccharidosis. Mol. Genet. Metab..

[26] Menkovic I., Lavoie P., Boutin M., Auray-Blais C. (2019). Distribution of heparan sulfate and dermatan sulfate in mucopolysaccharidosis type II mouse tissues pre- and post-enzyme-replacement therapy determined by UPLC-MS/MS. Bioanalusis.

[27] Colón C., Alvarez J.V., Castaño C., Gutierrez-Solana L.G., Marquez A.M., O’Callaghan M., Sánchez-Valverde F., Yeste C., Couce M.L. (2017). A selective screening program for the early detection of mucopolysaccharidosis. Medicine.

